# Queering the Mental Health Model

**DOI:** 10.1192/bjb.2019.45

**Published:** 2019-08

**Authors:** Andrew Camden

**Affiliations:** ST6 in General Adult Psychiatry, South London and the Maudsley NHS Foundation Trust, UK. email: Andrew.Camden@doctors.org.uk

I have been involved in a project run by the artist La JohnJoseph (JJ) on ‘Queering the Mental Health Model’. JJ's interest in the area was sparked by their experience visiting an ex-partner who was detained on a psychiatric ward, where ‘queerness’ often seemed to be conflated with illness by ward staff. JJ observed the difficulty of maintaining individuality in the confines of the ward environment; they noted that an attempt to do so was at times interpreted by staff as a sign of pathology.

JJ led 10 sessions over 12 months around the UK, to which people who self-identify as queer were invited to talk about their experience of psychiatric services. There was a general sense that people felt poorly understood outside specialist services, which are difficult to access; participants described a sense of having to fight their way through the system. There was, however, a multitude of positive feedback from people who had felt able to access a small number of appropriate specialist services.

‘Queer’ tends to be used now as an umbrella term to describe non-cis or non-heterosexual people. Historically derogatory, from the early 1990s, queer was reclaimed as a self-affirming term, originally by the gay rights movement. Queer challenges our tendency towards conventional categorisation into defined groups and helps remind us of individuality and the diversity of identity. As part of the LGBTQ community, it is known that people who self-identify as queer suffer higher rates of mental distress than the population at large.^1^ JJ's project will shed some light on the reasons for this.

There is a dearth of literature on this topic. A literature search by Reay House Library (based at Lambeth Hospital in London) on the ‘experience of psychiatric/mental health services of people who self-identify as queer’ found a total of 33 articles, of which four were by UK authors. None of these had a specific focus on people who identify as queer; they described experiences of the UK LGBTQ community in general. Two articles have an historical focus. Hughes *et al* have published two recent articles, one looking at the experiences of LGBTQ youth (ages 16–25) of suicidality and help-seeking^2^ and one on the perceptions and practice of mental health staff of this group.^1^ There is clearly scope to further examine the experience of psychiatric services of people who identify as queer, to help inform how these might better meet demand and reduce feelings of distress and marginalisation.

JJ's performance piece based on their experiences on the psychiatric ward, *A Generous Lover*, will tour the UK in September. There is further information at www.lajohnjoseph.com.
